# Brain and Cognitive Development in Adolescents with Anorexia Nervosa: A Systematic Review of fMRI Studies

**DOI:** 10.3390/nu11081907

**Published:** 2019-08-15

**Authors:** Gaia Olivo, Santino Gaudio, Helgi B. Schiöth

**Affiliations:** 1 Department of Neuroscience, Functional Pharmacology, Uppsala University, 751 24 Uppsala, Sweden; 2 Centre for Integrated Research (CIR), Area of Diagnostic Imaging, University “Campus Bio-Medico di Roma”, 00128 Rome, Italy; 3 Institute for Translational Medicine and Biotechnology, Sechenov First Moscow State Medical University, 119146 Moscow, Russia

**Keywords:** adolescents, adolescence, eating disorders, anorexia nervosa, fMRI, functional magnetic resonance imaging

## Abstract

Anorexia nervosa (AN) is an eating disorder often occurring in adolescence. AN has one of the highest mortality rates amongst psychiatric illnesses and is associated with medical complications and high risk for psychiatric comorbidities, persisting after treatment. Remission rates range from 23% to 33%. Moreover, weight recovery does not necessarily reflect cognitive recovery. This issue is of particular interest in adolescence, characterized by progressive changes in brain structure and functional circuitries, and fast cognitive development. We reviewed existing literature on fMRI studies in adolescents diagnosed with AN, following PRISMA guidelines. Eligible studies had to: (1) be written in English; (2) include only adolescent participants; and (3) use block-design fMRI. We propose a pathogenic model based on normal and AN-related neural and cognitive maturation during adolescence. We propose that underweight and delayed puberty—caused by genetic, environmental, and neurobehavioral factors—can affect brain and cognitive development and lead to impaired cognitive flexibility, which in turn sustains the perpetuation of aberrant behaviors in a vicious cycle. Moreover, greater punishment sensitivity causes a shift toward punishment-based learning, leading to greater anxiety and ultimately to excessive reappraisal over emotions. Treatments combining physiological and neurobehavioral rationales must be adopted to improve outcomes and prevent relapses.

## 1. Introduction

The lifetime prevalence of AN in females ranges from 0.5 to 2.2% [1,2], while is tenfold lower in males [3]. The onset often occurs during adolescence (13–18 years) [1]. Adolescent-onset AN seems to be associated with better remission rates [2]. Nonetheless, recovery can take several years [2], and the risk for relapse is particularly high during the first 16 months after treatment and increases with time [3,4,5]. AN has a mortality rate of 5–6%, estimated to be amongst the highest of all psychiatric illnesses [1]. Moreover, AN is associated with a trait-dependent high prevalence of comorbid psychiatric disorders, persisting after treatment and recovery [2]. The most common comorbidities are mood disorders, personality disorders, anxiety disorders, obsessive-compulsive disorders, and developmental disorders (e.g., autistic spectrum, attention-deficit hyperactivity disorder) [6]. These comorbidities can complicate treatment and need to be addressed in order to achieve a better outcome [6]. Furthermore, AN is also associated with several short-and long-term systemic consequences for the reproductive, cardiovascular, gastrointestinal, and skeletal systems, such as hypoestrogenism, decreased bone mass density, and consequent increased prevalence of osteopenia and osteoporosis, fertility problems, cardiac complications such as sinus bradycardia, prolonged QT interval on electrocardiography, arrhythmias, myocardial mass modification, and hypotension [7]. Thus, a prompt identification and treatment of AN is of utmost importance.

Family-based treatment (FBT) is currently the leading therapy for adolescent AN [8]. End-of-treatment remission rates in adolescent AN range from 23% to 33% [9,10], with only one-third still in remission at four-year follow-up [11]. Thus, there is a need for treatments with more precise and specific rationale, addressing not only physiological symptoms, but also cognitive and behavioral symptoms [10]. Enhanced cognitive behavioral therapy (CBT-E) has given promising results [12]. CBT-E is an individual-tailored treatment that targets the specific psychopathological mechanisms sustaining the eating disorder (ED) [12]. Indeed, weight-based recovery does not necessarily reflect broader cognitive recovery, as the complex twist of cognitive and affective symptoms characteristic of AN, including the fear of weight gain, body dissatisfaction, emotional dysregulation, and fear of calorie-dense foods, frequently persist after weight restoration [10]. Functional neuroimaging is a useful tool to investigate the neurobiological pathogenesis of psychiatric disorders. However, interpreting brain-imaging findings in adolescents is particularly challenging, as adolescence is characterized by remarkable brain plasticity, with fast and profound changes occurring in brain structure and functional circuitries. The adolescent brain is very sensitive to the influence of the genetic background and the environment, and several factors can affect brain development during this critical period, often with specific effects on certain brain areas or functional systems. Understanding the structural and functional brain maturation, and the role played by physiological and environmental factors, is thus crucial to interpret the alterations observed in AN.

### 1.1. Impact of Genetic and Environmental Factors on the Adolescent Brain 

Puberty has been associated with decreasing grey matter volume in several cortical areas, such as the frontal lobes and less consistently the temporal lobe, as well as in subcortical structures, such as the amygdala [13]. Thinning of the cortex during adolescence and early adulthood has been linked to increased myelination, reflective of maturation [14]. As for white matter, the density/volume increases with puberty and with hormonal levels, predominantly in the frontal regions and in cortico-subcortical projections [13]. Indeed, white and grey matter development seems to be overall synchronized [15] and frontal regions, involved in cognitive control, appear to be consistently associated with puberty and gonadal levels [13]. 

Cortical thickness seems to be also under genetic regulation [14]. Genes involved in synaptic function, dendrite development, and myelination exert a prominent regulatory role over cortical development, leading to different developmental trajectories for different brain regions [14]. During adolescence, there is a shift in the distribution of hub regions toward the frontal areas, and hubs in the associative cortices undergo a fast myelination process and cortical shrinkage between 14 and 21 years [15]. Thus, primary areas might experience a peak in genetic regulation over maturation during early adolescence and a decrease in late adolescence, while genetic influence is higher during late adolescence for associative cortices [14]. However, changes in grey matter volume in many structures seem to be non-linearly associated with age, particularly for deep grey matter structures [16,17], experiencing a linear volumetric decrease between 13–17 years and a slower decrease in the 18–27 years old period [18]. This pattern is consistent with the hypothesis of differential maturation period for different structures. Specifically, early maturation of the basal ganglia may relate to basic functions required for early development, such as motor initiation, learning, and reward-seeking, while the later maturation of the thalamus, hippocampus, and amygdala may reflect the need for more complex, integrated responses necessary for higher-order functions, such as emotional processing and memory consolidation [18]. 

Functional networks are also influenced by gonadal levels during puberty, particularly in terms of increased motivational processing in the ventral striatum and medial prefrontal cortex (PFC), and increased social-cognitive processes in the social brain network occurring with pubertal maturation [13]. Moreover, functional connectomes become more stable during adolescence. Long-range connections strengthen during development and short-range connections weaken [15]. Different networks also undergo selective modifications, with the default mode network (DMN) showing increasing inter- and intra-system connectivity and the sensorimotor network (SMN), on the other hand, becoming increasingly segregated from other systems [15]. Moreover, several other factors can affect brain development in this critical period, such as nutrition [19], history of stress during childhood [20], and psychological constructs [21].

### 1.2. Aim of Our Review

We aim to systematically review current literature concerning functional brain alterations in adolescents with AN and discuss the neurobehavioral circuits involved, in light of current knowledge regarding normal cognitive brain development, to better understand the deviations from this trajectory occurring in AN. Building on previous findings, we will identify the specific cognitive domains involved in the development of adolescent AN, with a specific focus on their implications for the pathogenesis and treatment of this disorder. Furthermore, we will discuss the limitations and specific issues in neuroimaging research on adolescent AN, and the role potentially played by confounding factors.

## 2. Methods

### 2.1. Search Strategy and Eligibility Criteria

PRISMA guidelines [22] were followed for the identification of eligible studies. The PRISMA checklist is reported in Table S1. Medline and Scopus were searched using the keywords “anorexia nervosa” AND (“fMRI” OR “functional magnetic resonance”) AND (“adolescence” OR “adolescents”) for journal articles published from inception up to March 12, 2019. Duplicates were discarded, and titles and abstracts were screened to determine the eligibility for the full-text screening stage. Eligible studies were additionally screened for references to identify studies not retrieved by the database search.

**Figure 1:**
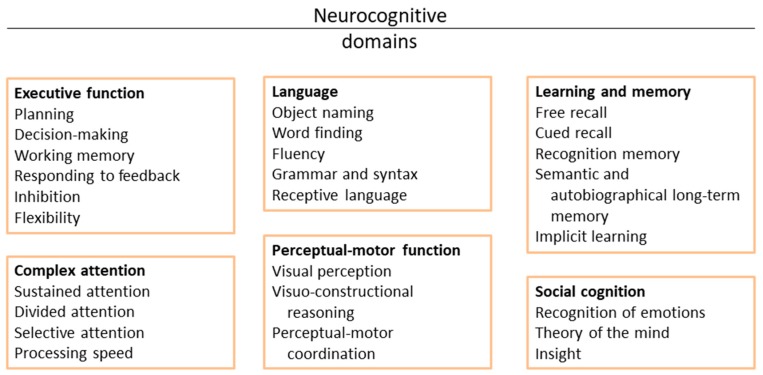
Cognitive domains and subdomains. The figure represents the classification of the cognitive domains and subdomains proposed by the DSM-5.

To be included in the review, studies were required to: (1) be written in English; (2) include only adolescent participants (10–19 years, as defined by the World Health Organization) with current or recovered AN; and (3) use fMRI with a block-design or task protocol to investigate neural activity. Resting-state fMRI studies were not considered in this review. Studies involving mixed samples of adolescents and young adults were only considered if the effect of age had been thoroughly investigated, by treating these ages as different samples, or by verifying the findings separately in the two ages. According to the classification of neurocognitive domains proposed by the DSM-5 [23] the papers were assigned to one of six neurocognitive domains: perceptual-motor learning, language, learning and memory, executive function, complex attention, and social cognition. Each paper was then further characterized based on the specific neurocognitive subdomain assessed (Figure 1). Reward processing was considered as a subdomain of the “learning and memory” category, given the key role played by reward processing in the learning process [24,25]. Stimuli specifically targeting ED-related psychopathology (body images, food images) were considered separately.

### 2.2. Quality Assessment

No standardized protocols are currently recommended for studies’ quality assessment. We thus followed the recent guidelines for neuroimaging research in EDs suggested by Frank et al., 2018 [26]. Such guidelines comprise 31 items, divided in six categories concerning respectively: development, demographic data, and illness state; effects of exercise, hydration status, binge eating, and purging, and malnutrition; stage of treatment; hormonal effects; comorbidity and medication; technical and statistical considerations, and study design. See supplementary material (Quality Assessment) for the full list of items and details on the scoring system. Due to the cross-sectional nature of most of the studies considered in the current review, item #6 (i.e., “Every effort should be made to describe level of recovery in a study sample and its relationship with brain findings”) was not considered. Additionally, items #21 (i.e., “If hormone levels are measured, similar to the recommendation to standardize nutritional status, the conditions under which hormones are assessed should be standardized and reported”) and #22 (i.e., “Other factors that influence the hormonal milieu should be considered”) were disregarded; these items concerned the reporting of the conditions used for hormonal measurements, which were not carried out in these studies. Each item was assigned a score ranging from 0 to 1 (see supplementary material (Quality Assessment) for the detailed information on the scoring system used). In their guidelines, Frank et al. [26] also assign a weight to each item, based on whether it is considered as desirable (1), strongly desirable (2), or essential (3). Thus, we have weighted the scores by multiplying the score on each item for the corresponding weight proposed by the guidelines (see supplementary material (Quality Assessment) for details regarding the weighting system). It has to be noted that there is no consensus over the minimum sample size required for fMRI studies [26]. However, we used the criterion proposed by Thirion et al. [27], as suggested by Frank et al. [26].

## 3. Results

**Figure 2:**
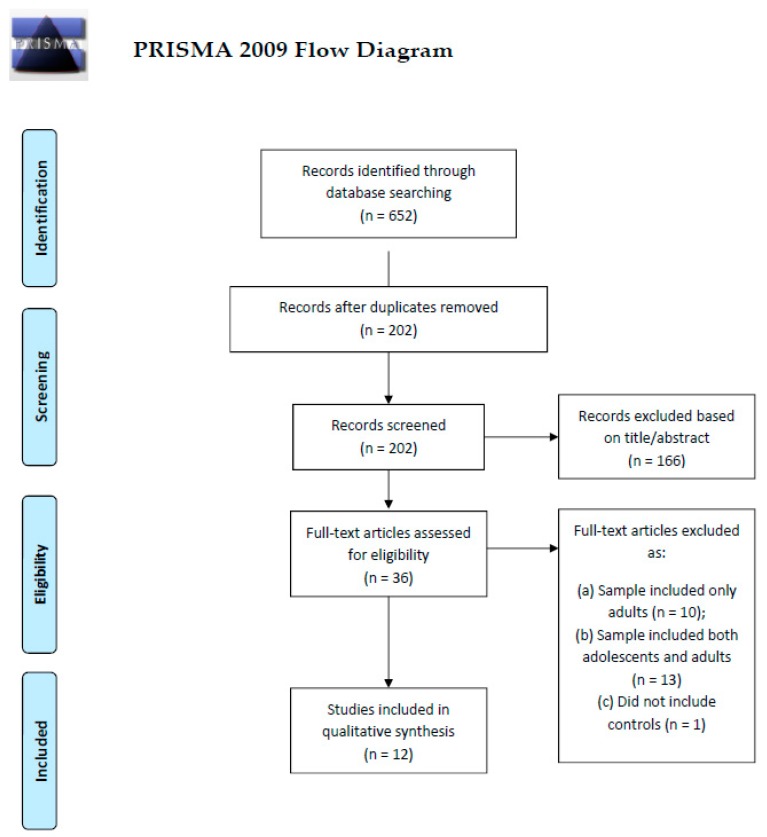
Flow-chart of study selection. The flow-chart reports the steps performed for the selection of studies to be included in the current review, according to PRISMA guidelines. Studies involving mixed samples of adolescents and young adults were only considered if they treated them as different samples, or verified the findings separately in the two age groups.

**Figure 3:**
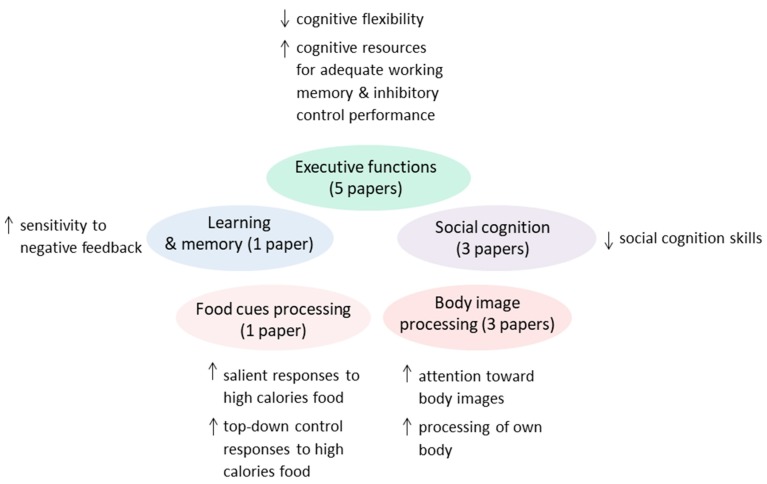
Main conclusions from the reviewed papers. The figure summarizes the main conclusions drawn from the authors of the reviewed papers (*n* = 12), relative to each cognitive domain. The findings are further discussed in the Results section.

After full-text screening, 12 studies were included in this review (Figure 2). In total, 170 patients (87 restrictive subtype, 18 binge-purging subtype, 65 unspecified) and 169 controls were included. The papers were relative to: executive functions (*n* = 5) [28,29,30,31,32], learning and memory (*n* = 1) [33], social cognition (*n* = 3) [34,35,36], ED-related stimuli (*n* = 4) [34,37,38,39]. One paper [34] reported both emotional and ED-related stimuli; the tasks are considered separately. Firk et al. [28] used an implicit learning (learning and memory) protocol, however the study aimed at exploring cognitive flexibility and was thus included in the executive function section. All the studies focused on acutely ill patients except for Xu et al. [36], focusing on weight-restored patients; the studies by Castro-Fornieles et al. [30] and Schulte-Ruther et al. [35] had a longitudinal design, and investigated patients in the acute and remitted state. The characteristics of the samples and main findings and conclusions from the papers are summarized in Table 1 and Figure 3. A detailed description of the findings relative to each cognitive domain is provided in the following paragraphs.

### 3.1. Executive Functions

Executive functions, also known as cognitive control, encompass a set of cognitive processes required for goal-directed behavior. Five studies assessed executive functions in adolescents with AN. Two studies specifically targeted cognitive flexibility [28,29]; one study, which also had a longitudinal assessment, targeted working memory [30]; two other studies examined inhibitory control [31,32] (Table 1, Figure 3).

Cognitive flexibility is the ability to switch cognitive processing strategies to adapt to unexpected changes in the environment. Two specific aspects of cognitive flexibility were investigated respectively by Firk et al. [28] and Hildebrandt et al. [29]: implicit learning, i.e., learning of complex information incidentally, without being aware of the learning process; and reversal learning, the ability of modifying a previously learnt stimulus-response association. Firk et al. reported impaired implicit learning in AN patients, associated with lower activity in ventral thalamic nuclei [28]. On the other hand, Hildebrandt et al. showed that although reversal learning abilities were not impaired in AN patients, the task required significantly more cognitive resources in AN, particularly in frontal areas, involved in decision making and cognitive control [29]. The same pattern was observed for working memory by Castro-Fornieles et al., who reported a normal working memory performance in AN patients associated with hyperactivity in temporal areas [30] using a 1-back task. During this task, the participants are shown a sequence of letters, and have to press a button when the letter on the screen is the same as the previous letter. This latter study also included an exploratory longitudinal evaluation of the patients, suggesting that neural activity in decision-making areas is dependent upon weight status and depressive symptomatology [30].

Similar results were also found concerning inhibitory control. Inhibitory control represents the ability of an individual to inhibit their impulsive or habitual responses to a stimulus in order to select a more appropriate goal-directed behavior. Two different tasks, the Go-NoGo and the stop-signal task (SST), were used in the studies targeting inhibitory control. The Go-NoGo task requires the participants to respond (e.g., by pressing a button) to certain stimuli (Go stimuli), and to not respond (e.g., not pressing the button) to other specific stimuli (NoGo stimuli). In the SST, the participant is shown an arrow pointing either left or right, and has to indicate the direction in which the arrow points by selecting one of two buttons. When an audio tone is presented together with the visual stimulus, the participant has to withhold responding. Lock et al. reported a correlation between activity in brain areas related to visual attention and visual working memory and successful inhibitory control during a Go-NoGo task in AN patients [31]. However, patients and controls did not differ on either task performance or brain activity [31]. Using a stop-signal task, Wierenga et al. also reported no differences in accuracy between patients and controls, however patients tended not to slow down their response time after errors, showing a somewhat impaired monitoring ability [32]. Patients also had decreased activity in frontal areas compared with controls. Lock et al. [31] and Wierenga et al. [32] focused on the restrictive subtype of AN, while no information were available concerning the subtype of AN for the other studies [28,29,30]. Overall, these studies indicate impaired cognitive flexibility in AN adolescent patients; on the other hand, achieving an effective performance on working memory and inhibitory control tasks, though possible, requires nonetheless significantly higher cognitive load compared with controls.

### 3.2. Learning and Memory

The only study falling into the “learning and memory” category specifically aimed at investigating reward processing in AN patients with a restrictive subtype, reporting a normal reward sensitivity but an exaggerated sensitivity to punishment in patients compared with controls during an action-outcome learning task [33] (Table 2, Figure 3). Specifically, a monetary guessing task was used, where the participants was shown a down-facing card and had to guess whether the hidden number was less than or greater than five; according to their guess, they could either win or lose money. Feedback was given after each trial. Patients were more sensitive to negative feedback when learning from experience. Neural activity in the caudate nucleus and cognitive cingulate cortex was higher in AN patients during punishment processing compared with controls [33]. 

### 3.3. Social Cognition

Social cognition focuses on the role that cognitive processes play in social interactions, by studying how people process, store, and apply information about other people and social situations. Three studies investigated social cognition in mostly restrictive AN, one with a cross-sectional design [34] and two with a longitudinal design [35,36] (Table 2). Horndasch et al. investigated emotional processing in AN, although the AN subtype was not reported [34]. The participants were shown negative, neutral, or positive stimuli, and high calorie and low calorie foods. The food-specific results will be described in the ED-related section (see below). The rating of the emotional stimuli was not different from that of controls irrespective of the type of stimuli shown, however patients exhibited lower neural activity in response to both positive and negative stimuli in several cerebellar, striatal, and cortical areas [34]. Only the medial prefrontal cortex had higher activity in patients when viewing neutral and positive stimuli. Overall, patients seemed therefore to exhibit lower responses to all emotional stimuli [34] (Table 1, Figure 3). 

Schulte-Ruther et al. [35] used a social attribution task, in which the participants were shown a video of objects engaging in social behaviors and a video of objects moving randomly, and had to infer social relationships between objects. Patients could correctly attribute social relations, however they showed decreased activity in temporal regions involved in social cognition compared with controls [35]. These alterations persisted after treatment and weight restoration, and could predict treatment outcome [35] (Table 2, Figure 3). Xu et al. [36] yielded similar results using a different task, called the social identity V2 task, in weight-restored patients. In this task, participants were asked to read statements related to themselves or friends and decide whether they agreed or not. Patients and controls did not differ on neural activity [36]. However, activity in the posterior cingulate cortex could predict treatment outcome within patients. In particular, those who had higher activity when reading self-evaluating sentences written by friends rather than by themselves, had a worse treatment outcome [36] (Table 2, Figure 3). Overall, these studies are consistent in suggesting impaired social cognition skills in AN patients.

### 3.4. ED-Related Stimuli

Studies using ED-related stimuli have targeted either body disturbances or food bias. Two studies on body image perception have focused on mostly restrictive AN [37,39], while another exploratory study only involved three patients of unknown subtype [38]. Food-specific responses were only investigated by Horndasch et al. [34], for which information regarding the AN subtype was not available (Table 2, Figure 3).

Fladung et al. [37] showed images of underweight, normal weight and overweight stranger females to the participants. The authors aimed at demonstrating that AN shares the same neural circuitry as addiction, and were thus aiming to elicit reward responses with this task [37]. However, body image disturbances and body shape concerns are key criteria for the diagnosis of AN, thus we decided to include this study in the ED-related task. AN patients rated underweighted women higher than controls. Moreover, patients had higher neural activity when processing underweight body images compared with controls specifically in the ventral striatum, while controls had higher activity when processing normal weight images, supporting the hypothesis that AN might be considered as a starvation-dependent disorder [37]. Interestingly, within patients, no selective neural responses to normal weight or underweight stimuli could be observed, while controls had higher activity when processing normal weight stimuli compared to underweight [37].

Seeger et al. [38] and Wagner et al. [39] used the same protocol, consisting in the presentation of distorted images of the participants’ own body or of other women’s bodies, or neutral control images. The pilot on three patients carried out by Seeger et al. showed higher activity in the amygdala and fusiform gyrus in patients when viewing their own distorted body, suggesting aversive reactions in response to their own weight-increased body shape [38]. However, this finding was not confirmed by a later study by Wagner et al. [39]. In this study, both patients and controls had higher prefrontal activity when viewing own body images compared to neutral, however the activity was higher in patients than controls [39]. Moreover, the activity in patients was also higher when viewing other women’s bodies compared to neutral images, while no differences were observed in controls [39]. Furthermore, the inferior parietal lobule was more active in patients when viewing own body images compared to other women’s or neutral pictures, while no difference were observed in this area in controls [39]. Patients therefore showed an unspecific attentive response to body stimuli, and an greater visuo-spatial processing of their own body shape [39]. The above-described studies investigating body image processing in AN are therefore consistent in reporting altered body perception in AN, with increased attention toward body-related cues.

Horndasch et al. showed participants high and low calorie food pictures [34]. AN patients and controls rated low calorie foods as pleasant, while AN patients gave lower rating to high calorie foods compared with controls. Neural activity was higher in patients in inferior frontal, prefrontal and insular cortices and decreased in the cerebellum compared with controls in response to high calorie food pictures, while the pattern of activity was reversed in response to low calorie food images [34]. Thus, patients showed higher activity in top-down (cognitive control) and bottom-up (salience and affective responses) regions compared with controls [34].

### 3.5. Quality Assessment of the Studies

The overall quality of the studies was not very high, with a mean quality score (QS) of 29.5 on a possible range of 0–70 (Table 2). The scores were especially low on the “development, demographic data, and illness state” scale. This was mainly due to the lack of testing for body mass index (BMI) influence on neural activity, and to the lack of information concerning handedness and ethnicity. The effects of exercise, hydration status, binge eating and purging, and malnutrition on brain activity were also overlooked, as well as the effect of the hormonal status. Although we can assume that patients diagnosed with full-threshold AN according to DSM-IV mostly had second amenorrhea, conditions such as premenarche or the use of contraceptive pills cannot be ruled out in either patients or controls unless explicitly stated in the methods. Based on this overview, we encourage authors to be as detailed as possible in characterizing their cohorts. Moreover, while most of the studies seemed to have good quality in terms of technical and statistical considerations and study design, it has to be noticed that none of the studies tested the data for normality of the distribution. Additionally, sample size is not included in the criteria proposed by Frank et al. [26], used for this quality assessment. As pointed out in the guidelines, there is no consensus over the minimum sample size required for fMRI studies. This is a major drawback, as neuroimaging studies—particularly if focusing on adolescents—are often carried out on very small samples. However, the study by Thirion et al. [27] is mentioned, which suggest that a sample size of at least 20 participants is necessary for reliable fMRI studies. Thus, we considered studies to have an adequate sample size if at least one of the groups (either patients or controls) included 20 or more individuals [27]. Only three studies (Firk et al. [28], Schulte-Ruther et al. [35], and Xu et al. [36]) had an adequate sample size according to this criterion. Thus, studies with larger sample sizes are desirable to verify the validity of our conclusions.

On the other hand, most of the studies appropriately reported information concerning the assessment of comorbidity and medication use, and the stage of treatment. In the study conducted by Frick et al. [28], two patients were on medications; although the duration of medication use was not reported, an additional confirmatory analysis was run by excluding these two subjects and yielded the same results. Thus, item #26 (i.e., “Ideally, medication use should be stable for four to five half-lives before brain imaging to accomplish at least steady state conditions”) was considered as positive.

## 4. Discussion

We reviewed 12 fMRI studies investigating different cognitive domains in adolescent patients with AN compared with controls. We discuss the findings in AN in light of current knowledge on the development of specific cognitive domains during adolescence, in order to better understand the deviations from this trajectory occurring in AN. We then provide a theoretical framework to delineate a tentative pathogenic model for the development of adolescent AN, and its implications for treatment. Furthermore, we discuss the limitations and specific issues in neuroimaging research on adolescent AN, and the role potentially played by confounding factors.

### 4.1. Executive Functions in Healthy and Anorectic Adolescents

Cognitive flexibility was reported to be impaired in AN patients [28,29]. In particular, the performance on implicit learning was lower in AN than controls, and associated with lower activity in the ventral anterior and ventral lateral thalamic nuclei [28]. On the other hand, patients performed as good as controls on explicit learning, specifically on a reversal learning task, but required a greater engagement of the frontal, top-down control regions in order to achieve such result [29]. Implicit and explicit learning differ in that implicit learning occurs in an incidental manner, without awareness of the process itself and, as such, the produced knowledge is not consciously accessible. Lower thalamic activity during implicit learning in AN has been associated with a lack of top-down priming, and seems to be a state-dependent alteration subsiding with prompt treatment [28]. The thalamus matures relatively late during adolescence [18] by overall expanding its volume [40], though the ventral anterior and lateral nuclei specifically tend to contract with age [40]. These nuclei are the most interconnected with prefrontal and parietal regions [40], and have been proposed as key structures for the risk of depression and risk-taking behaviors in adolescence [40]. Thus, AN patients might exhibit a lower or slower maturation of the thalamus with subsequent impairment of the implicit learning processes and relative cognitive flexibility. On the other hand, the compensatory greater demand on frontal top-down circuitry might be sufficient to overcome the impairment in cognitive flexibility during explicit (conscious) learning, and lead to an adequate performance. Indeed, implicit and explicit learning engage different neural networks, as frontal and parietal structures mediate the attentional and episodic aspects of explicit learning, while direct fronto-striatal connections subserve implicit learning [41]. Differences in quality between these studies have also to be noted, with a QS of 31.5 for the study on implicit learning by Firk at al. [28], while the study on explicit learning by Hildebrandt et al. [29] barely reached a QS of 12.5, well below the mean of the included studies. 

The study from Castro-Fornieles et al. had, on the other hand, overall good quality with a QS of 35.5, and showed that working memory performance also required higher cognitive load in several temporal and parietal areas in adolescents with AN compared with controls, in order to achieve the same results [30]. Indeed, higher activity in frontal and parietal areas is associated with working memory performance [42]. Moreover, within patients, the cognitive load required increased with increasing depressive symptomatology, and decreasing BMI. In particular, the depressive symptomatology correlated with the activity in areas involved in visual processing, such as the angular and fusiform gyrus. Interestingly, working memory has been reported to rely on executive regions in childhood, while adolescence is characterized by the recruitment of specialized visual and parietal areas [43]. On the other hand, BMI negatively correlated with activity in frontal areas involved in cognitive control. Frontal lobes tend to mature later than other structures during adolescence, and become progressively dominant in planning, organizing, and regulating thought and behavior [44]. The maturity of fronto-striatal connections seems to be particularly relevant in such regard, with a prominent role played by the caudate nucleus [42]. Thus, low BMI and depressive symptomatology in AN patients are associated with lower efficiency of the working memory system, though the pattern of development follows the typical developmental trajectory. The reduction in efficiency was however a state-dependent alteration, which subsided after weight restoration and treatment [30]. This might be at least partly related to the normalization of hormonal levels occurring with recovery, as the maturation of frontal regions is associated with puberty and gonadal levels [13].

The two studies exploring inhibitory control in adolescents with AN, on the other hand, point to somewhat conflicting interpretations. In fact, Lock et al. [31] report that patients can achieve the same level of inhibitory control as healthy controls, however they need more cognitive resource to do so. In particular, they were found to have a higher recruitment of visual attention and visual working memory areas, such as the inferior parietal cortex, precuneus, and posterior cingulate cortex (PCC). On the other hand, Wierenga et al. [32] report lower activity in AN patients compared with controls in middle frontal gyrus (MFG), ACC, and PCC, indicating that patients need less cognitive resources for error monitoring. Thus, while the results from both Lock et al. [31] and Wierenga et al. [32] agree on the achievement of a successful cognitive control in adolescents with AN, future studies will have to verify whether greater recruitment of relevant brain regions is necessary to this outcome. It is worth noting that both studies had QS above the average of the included studies (QS = 33.25 for Lock et al. [31] and QS = 36.75 for Wierenga et al. [32])

In healthy individuals, inhibitory control and decision-making progressively improve from childhood to mid-adolescence with remarkable inter-individual variability, as demonstrated by delayed-discounting tasks [45]. Delayed-discounting is the tendency to prefer to wait for a larger, delayed reward over a smaller but immediate reward, and reflects cognitive control and decision-making capability. In healthy adolescents, delayed-discounting is associated with higher connectivity between the prefrontal cortex (PFC) and the dorsal ACC (cognitive control regions), and with lower connectivity between the pallidum and the PCC (evaluation regions) [45]. In particular, a functioning cognitive control seems to depend on the quality of connections between the PFC and striatum, such that stronger connectivity can predict less impulsive choices after a two years follow-up [46]. The development of fronto-striatal white matter follows a non-linear trajectory, with relative fast development during childhood and early adulthood and little change during mid-adolescence [47]. However, several factors can influence inhibitory control development. Nutrition, for example, can affect the maturation of the dorsal ACC. In particular, it has been suggested that a lower Omega-3 intake during adolescence might lead to inefficient metabolism in the dorsal ACC, or to impaired pruning of superfluous axonal connections, leading to higher activity [19]. Moreover, cognitive control seems also to be negatively affected by a history of stressful experiences at an earlier time in life, leading to poorer interpersonal relationship quality during adolescence [20]. Furthermore, the personal conception that adolescents have of their own age is associated with activity changes in the frontal regions during the transition from middle to high school, reflecting the influence of psychological adjustment during this shift [21]. In particular, subjects who conceived adolescence as a period of “rebellion” toward family rules showed a greater increase in frontal activity when shifting from middle to high school, coupled to higher risk-taking behavior [21]. 

Overall, AN adolescents exhibit the same pattern of maturation and functional specialization of thalamic nuclei, frontal areas, and fronto-striatal connections as healthy adolescents. However, they seem to have a somewhat slower development of these brain regions, leading to impaired cognitive flexibility and working memory, partially overcome by a compensatory higher demand on top-down frontal areas. These alterations are likely to be state-dependent and influenced by low BMI and altered pubertal status, and can be rescued with prompt treatment and weight restoration. On the other hand, the impairment in inhibitory control seems to be more complex, and probably influenced by other factors such as childhood stress, social conceptions, as well as nutrition and puberty.

### 4.2. Learning and Reward Processing

One study investigated reward processing in adolescents with AN by using a reward-based learning task [33] with a good QS of 35.75, and reported normal behavioral performance in AN, although they exhibited higher neural responses to negative feedback. In particular, the posterior caudate and cognitive cingulate cortex were more responsive to negative feedback (punishment) in AN compared with controls. When comparing positive to negative feedback within groups, the putamen and motor cingulate cortex were more responsive to positive rather than negative feedback in controls, while no differences in activity based on feedback were observed in AN patients [33]. 

Interestingly, the caudate is more active in response to negative rather than positive feedback also in healthy adolescents [48]. The higher activity in the caudate of AN patients in response to negative feedback was proposed to bias their ability to proportion reward and punishment when learning from experience [33]. However, while the responsivity to negative vs. positive feedback in the caudate remains stable from childhood to adulthood (8–29 years), the selectivity toward negative feedback increases with age [48]. As AN patients were on average one year older than controls in the study by Bischoff-Grethe et al. [33], that might have partially driven the results. Nonetheless, higher caudate activity in adolescence has been associated with better current and future learning performance, as it supposedly reflects higher sensitivity to the informative value of feedback [48]. The striatum is particularly engaged when learning new rules rather than applying known rules, and its responsiveness to feedback peaks around 17–20 years, suggesting that adaptive learning occurs during late-adolescence [48]. This is also reflected by behavioral performance [48], supporting the hypothesis that AN adolescents might create stimulus-learning associations in response to negative outcome.

The reward system is influenced by developmental differences in self-reported motivation to rewards seeking (trait-dependent) and the immediate pleasure from winning (state-dependent) [49]. The marked responsivity to reward in mid-adolescents might be related to the peculiarly high dopamine release during this period [49]. However, pubertal hormones also play a role, such that higher testosterone levels contribute to enhance reward sensitivity [50]. Worth noticing, AN patients generally have reduced testosterone levels [51,52], which might contribute to the altered development of reward-sensitive regions. This might shift the learning process toward negative feedback. Moreover, family and social environment contribute to the development of the reward system. In particular, low parental warmth in early adolescence can predict higher prefrontal and striatal responses to reward in mid-adolescence [53]. In the study by Casement et al., low parental warmth was reported by the parents, and measured how often they wished their daughters would leave them alone [53]. It is worth noting that AN patients, particularly the restrictive subtype studied by Bischoff-Grethe et al. [33], generally come from enmeshed [54] and over-controlling [55] families, which would reflect high parental warmth as measured by Casement et al. [53], potentially contributing to their hypo-responsivity to rewards. On the same line, greater peer victimization, often occurring in AN and other eating-disordered patients [56], was associated with lower reward responsivity, though at a lesser extent [33].

### 4.3. Social Cognition

Different brain areas underlie self- vs. others-evaluation in healthy adolescents, with the temporo-parietal junction more responsive to others and the medial PFC more responsive toward self-evaluation [57]. Interestingly, Schulte-Ruther et al. (QS = 31.5) reported that, despite AN patients performed as well as controls on a social attribution task, they had lower activity in temporal areas compared with controls when viewing social vs. non-social movies, partially persisting after treatment and correlating with worse treatment outcome [35]. This might reflect a lesser engagement of the temporal areas involved in social processing, which might be related to a trait-dependent characteristic of AN patients, i.e., poor awareness of emotions (alexithymia) [58]. Indeed, greater activity in the temporo-parietal junction in adolescents reflects more advanced perspective-taking [59].

On the other hand, Xu et al. [36] (QS = 30.25) reported a correlation in AN patients between prefontal-cingular activity and body shape concerns and anxiety in a social identity task. In healthy adolescents, the selective responsiveness to self-evaluation of the medial PFC increases with age, particularly concerning the evaluation of one’s self from a social perspective, and it is thought to reflect the increasing importance that adolescents give to peers and social status [57,59]. In fact, activity in the medial PFC is also elicited by anticipation of positive social feedback, i.e., when individuals expect to be liked by their peers [59]. This suggests that AN patients, compared with controls, might get more anxious toward peers evaluation and how they think their friends perceive them. It is worth noting that while no differences were observed in the self- vs. friends-processing between AN patients and controls, higher activity in the posterior cingulate cortex in response to friend-related stimuli compared with self-related stimuli was related to treatment success [36]. Activity in the PCC in adolescents has been attributed to emotional reappraisal, the capacity of controlling emotions [60]. Thus, reappraisal strategies might help patients controlling their anxiety and reactions to peers’ behaviors. This is also supported by the role of the PCC in processing social exclusion feelings [61,62]. These findings suggest lower neural processing of social-cues in AN patients, coupled to higher social-related anxiety. In particular, AN patients are characterized by lower processing of social cues, maybe reflective of altered development of areas involved in social cognition, leading to impaired empathy and perspective-taking abilities [36]. They also show higher tendency toward social anxiety and enhanced activity in areas related to the emotional reappraisal and the processing of social exclusion feelings [36]. As these aspects are related to treatment outcome, they might be a core neural feature of AN.

The medial prefrontal cortex is also a key structure for emotional processing, and was reported by Horndasch et al. [34] (QS = 29) to be more active in AN patients compared with controls when viewing neutral or positive, though not negative, stimuli [34]. Medial prefrontal cortex has also been reported to be active during emotional reappraisal [60], however its activity seems to be dependent also on personality traits. Shy individuals show a blunted decrease in medial prefrontal cortex connectivity toward the precuneus compared with less shy individuals [63]. Overall, however, AN patients exhibited lower neural processing of emotional stimuli compared with controls, though they did not differ on the ratings of emotional stimuli [34]. Specifically, patients with AN had lower activity in the precuneus and hippocampus in response to positive stimuli [34]. In healthy adolescents, activity in the precuneus and hippocampus in response to emotional faces is associated with greater engagement of the sympathetic nervous system activity, suggesting that affective processing elicited in the precuneus and hippocampus can trigger peripheral arousal and stress responses [64]. This suggests that positive emotional stimuli are less efficient in engaging arousal responses in AN patients compared with controls, in accordance with the abovementioned lower reward-responsiveness in AN patients compared with controls. Moreover, compared with controls, AN had lower activity in the striatum, ACC, and inferior frontal gyrus in response to both negative and positive stimuli, and lower cerebellar activity in all conditions [34]. Activity in the inferior frontal gyrus is consistently elicited by either happy (positive) or fearful (negative) faces in healthy adolescents [65], suggesting an overall impairment in emotional processing in AN patients regardless of stimulus valence. This is also supported by the lower activity observed in these patients in the striatum, as activity in the striatum underlying emotional regulation has been reported to increase during adolescence in healthy individuals [66]. Interestingly, parents’ negative emotion regulation abilities have also been reported to influence their sons/daughters’ emotional responses [67]. However, it has to be noted that the QS of the study by Horndasch et al. [34] was not very high, calling for cautiousness in interpreting their results.

### 4.4. ED-Related Stimuli

The same study from Horndasch et al. [34] showed that arousal responses to food images showed a different pattern than that observed for general emotional stimuli in AN adolescents. Patients gave lower ratings to high-calorie foods compared with controls, while no differences were observed in the ratings of low-calorie foods [34]. High-calorie food elicited higher inferior frontal gyrus, medial prefrontal cortex and anterior insula activity, and less cerebellar activity in patients compared with controls, while low-calorie food elicited lower activity in the medial prefrontal cortex and parietal lobe [34]. Worth noticing, there was a mismatch between the ratings of foods and neural activity in patients. In fact, despite patients rated high-calorie foods lower than controls, high-calorie foods still elicited a greater response in patients compared with controls in the anterior insula and in the inferior frontal gyrus. Activity in the anterior insula is elicited by all the steps of food consumptions (seeing, smelling, tasting, and rewarding/hedonic value attribution) [68], while the inferior frontal gyrus responds to emotionally salient stimuli [34,65]. However, this was paralleled by greater activation of the medial prefrontal gyrus, involved in emotional reappraisal strategies [60]. Patients might therefore perceive high-calorie foods as more salient and emotionally relevant compared with controls, but they seem to react to this heightened arousal by adopting effective reappraisal strategies, ultimately leading to lower ratings of high-calorie foods. In further support of this hypothesis, reappraisal of craving for unhealthy foods is associated with both decreased self-reported desire for the food and enhanced activity in both the inferior frontal gyrus and anterior insula in healthy adolescents [69], mirroring the neurobehavioral pattern exhibited by neural activity in AN patients. Moreover, the activity in the medial prefrontal cortex was lower, rather than higher, in patients compared with controls when viewing low-calorie food images.

Concerning body perception, the ventral striatum was found to be more active in response to underweight stimuli in AN patients compared with controls, while it was more active in controls compared with patients in response to normal-weight stimuli [37]. This was considered by the authors as a sign that, in AN patients, underweight body stimuli can engage the same circuitry involved in addictions. Thus, they suggest that AN might be considered as a starvation-dependent disorder. Interestingly, the striatum undergoes dynamic changes in dopamine receptors density during adolescence [70]. These changes contribute to morphological maturation of the striatum, and reflect the development of association learning abilities and cognitive flexibility [70]. Moreover, the plasticity of the dopaminergic system during development poses the risk for heightened sensitivity to rewarding stimuli. However, it is worth noticing that, within groups, controls were found to have higher striatal reactivity to normal-weight pictures compared to underweight images, while patients could not distinguish between the two conditions. Thus, the between-group difference might also have been driven by greater variability in response in controls, and a stable response in patients. This would suggest that, while controls do find normal weight images to be more appealing, patients’ reward responses are not engaged. Moreover, prefrontal activity was related to body processing in AN patients. In particular, prefrontal activity was elicited by viewing own body compared with neutral images in controls, and by viewing both own and others’ body compared with neutral images in patients, suggesting a general attentive bias toward body images in patients [39]. The development of the dopaminergic system in the prefrontal cortex during adolescence mirrors that of the striatum [70]. In particular, striatal bursts of activity in response to salient stimuli result in the recruitment of the prefrontal regions, in order to build adequate self-control models [70]. The prefrontal cortex has a key role in decision-making and inhibitory control, and while its activity peaks in adolescence, its maturation is slower compared with that of the striatum, leading to a lack of self-control in early phases [71]. Moreover, as discussed above, prefrontal cortex becomes increasingly responsive to self-evaluation rather than other-evaluation with age in healthy adolescents [57,59]. Thus, AN patients seem to have a slower maturation of the prefrontal regulatory circuitry. Alternatively, the recruitment of prefrontal regions might be due to greater need for inhibitory control in response to body viewing, as also supported by findings from Xu et al., reporting a correlation between prefontal-cingular activity and body shape concerns and anxiety in AN patients [36].

Patients also showed higher activity in the inferior parietal lobule when viewing their own body images compared to other women’s bodies or neutral shapes, while this difference was not observed in controls [39]. This is somewhat unexpected, as specialization toward self- vs. other-face recognition in the right IPL has been reported to occur in healthy adolescents [72]. In AN patients, this seems to point toward an earlier recruitment of this regions in self- vs. others-related tasks. This is in favor of the hypothesis proposed by Wagner et al., that AN patients might have an attentional bias towards bodies in general, and an increased processing of their own body [39]. Taken together, the findings from the two studies on body perception suggest that: (1) patients perceive underweight bodies as appealing as normal weight; (2) their attentional circuitry has a unspecific responsiveness to body images, maybe due to higher body shape concerns; and (3) their own body processing abilities are developing faster or are enhanced compared to controls. However, while the study by Wierenga et al. [32] had a QS of 30, the study by Fladung et al. [37] had a very low QS of 18, and the study by Seeger et al. [38], despite having a somewhat good QS of 30, only included three individuals. Thus, findings concerning body perception have to be interpreted cautiously.

### 4.5. Pathogenic Model

Several pathogenic models for AN have been proposed in recent years. Grzelak et al. have outlined the neurochemical, emotional, and cognitive factors underlying the disordered eating behavior in AN [73], stressing the need to understand the interrelation between these factors to attain better treatment outcomes. An attempt at proposing a more integrate model has been made by Munro et al. [74]. They review and build on previous models to provide a framework for the maintenance of the disorder and associated treatment resistance, with a focus on the role of self-fear and inadequacy feelings. In particular, they propose that starvation might represent a maladaptive physiological mechanism for feelings regulation that emerges in order to manage uncomfortable emotional and physical feelings [74]. This leads to a short-term rewarding feeling of safety and self-acceptance, but results in long-term maladaptive social behavior, characterized by insecure inter-personal relationships, lack of trust, social isolation, and adoption of anorexia as an identity [74]. However, as the authors point out, their model does not attempt to explain the development of the disorder [74], but rather than physio-psychological factors sustaining the maladaptive behavior.

**Figure 4:**
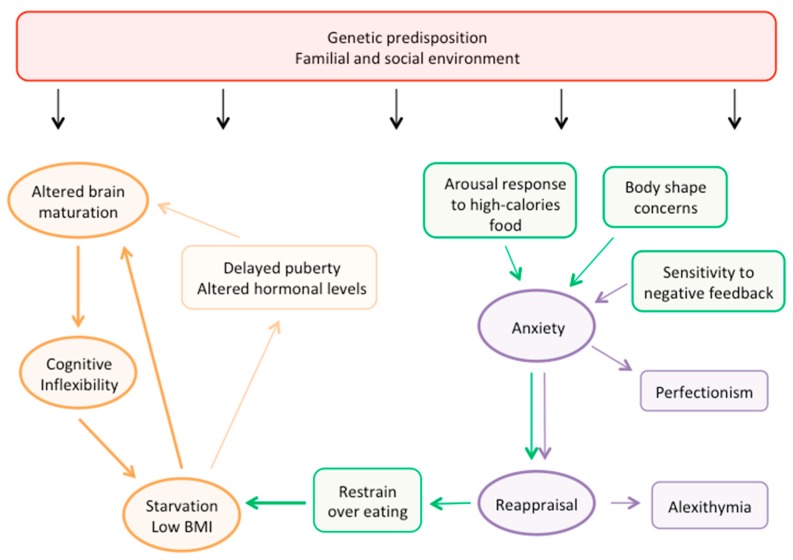
Pathogenic model of adolescent AN. The figure summarizes the pathogenic model of adolescent AN we propose, based on current fMRI findings in healthy and anorectic patients.

We propose an integrated pathogenic model specifically focused on adolescent AN, based on current knowledge of normal cognitive development and brain maturation patterns in adolescence (Figure 4). According to our proposed model, a major role in the risk for developing AN is likely to be played by genetics, with contribution from the familial and social environments.

In this framework, genetic predisposition might act through a dysregulation of the brain structural and functional development, leading to one of the core features of AN: cognitive inflexibility. Moreover, together with environmental factors such as early life stressors and parental relationship, the genetic background plays a role in determining specific psychological risk-traits in the individual, such as higher punishment sensitivity and more attention toward body-related stimuli and concerns. Body shape concerns and body-related anxiety ultimately lead to starvation and low BMI. Underweight causes a disruption in hormonal regulation and normal puberty trajectory, and both underweight and hormonal changes can directly impact brain maturation. Consequently, the deviation from normal brain development, and particularly the impairment in cognitive flexibility, sustain the maintenance of aberrant behaviors, and a vicious cycle is established, perpetuating restraint over eating and other AN-related behaviors. Consistent with this model, habitual behaviors have been reported to be more important than conscious cognitive control in sustaining food restriction in these patients [75]. On the other hand, cognitive control might play a key role in the impairment of social skills. Indeed, social and familial factors, together with the altered brain functional maturity, lead to the higher punishment sensitivity typical of AN adolescents, which causes a shift toward punishment-based learning. This translates in higher anxiety toward social stimuli, and ultimately in the adoption of reappraisal strategies to control the excess in negative emotions. In social life, this leads to one of the typical features of AN, alexithymia. In a broader perspective, reappraisal strategies might be used to reduce anxiety related to different types of stimuli and ultimately to food stimuli as well, that, by eliciting arousal, can jeopardize the maintenance of the underweight status.

### 4.6. Implications for Treatment

Family-based treatment (FBT) is currently the leading therapy for adolescent AN [8], however enhanced cognitive behavioral therapy (CBT-E), an individually-tailored treatment that targets the specific psychopathological mechanisms sustaining the ED, has also given promising results [12]. Nonetheless, the risk for relapse, particularly in the first 12–16 months after treatment, is still high in adolescents with AN [3,4,5], and was reported to be as high as 85% in some studies [5]. The risk for relapse increases with time, and long-term planning for relapse prevention should be made for all patients [4]. According to our proposed model, a combined approach might improve long-term treatment success and relapse prevention. Indeed, familial and social environments might have a leading role in the first stages of AN development, but once the process is started, it is likely to be sustained by the deficits in cognitive flexibility, aberrant association-learning processes, and excessive reappraisal strategies. Cognitive training to enhance strategy-shifting abilities could help improving cognitive flexibility; however, it should be coupled to the re-routing of the learning process toward adequate reward processing to facilitate the formation of beneficial strategy-outcome associations. Moreover, anxiety management strategies might help reducing cognitive reappraisal even in patients where anxiety problems are not directly evident at clinical observation. Schools should also be sensitized to detected difficulties in socialization, and for kids who consume their meals in school, an early detection of unhealthy eating behavior might help prevent the establishment of the vicious cycle leading to altered brain maturation and cognitive inflexibility.

### 4.7. Limitation of Current Research

Few studies addressed task-related functional brain alterations in adolescents with AN, so that only 12 studies could be included in the current review. Moreover, no studies have investigated the “language”, “perceptual-motor function”, and “complex attention” domains, leaving an important gap in our knowledge. Most of the studies had small sample sizes, thus conclusions are still preliminary. However, the studies were overall quite homogeneous in terms of gender (mostly females), AN subtype (mostly restrictive), age of the individuals included, and comorbidity status. Nonetheless, most of studies overlooked reporting fundamental characteristics of the patients, in terms of physical activity habits, nutritional status, and recent weight gain or loss. As all these factors can influence brain development in such a delicate period, reporting as many information as possible is encouraged. Furthermore, a more complete assessment of the familial environment might be useful to report along with neuroimaging data, in order to further assess the complex inter-relation between genetics, environment, and brain circuitry in leading to AN development and influencing treatment success.

## 5. Conclusions

We reviewed 12 studies using fMRI to investigate different cognitive domains in adolescents with AN, and critically interpreted the results in light of current knowledge concerning normal brain development during adolescence. AN adolescents exhibited altered maturation of the executive network, leading to impaired cognitive flexibility and working memory, partially overcome by a compensatory higher demand on top-down frontal areas. Low BMI and altered pubertal status were key factors in leading to executive functions impairment, and prompt treatment and weight restoration seem to be effectively rescue such alterations. On the other hand, the impairment in inhibitory control seems to be more complex, and probably influenced by other factors such as childhood stress, social conceptions, as well as nutrition and puberty. Moreover, AN adolescents have lower neural processing of social cues, reflective of a lack of empathy and perspective-taking abilities (i.e., alexithymia), and higher tendency toward social anxiety and emotional reappraisal. Social, familial, and genetic factors are likely to interact in determining the risk of developing AN. Cognitive inflexibility and enhanced sensitivity to punishment, leading to negative-feedback based association learning, might be particularly important in sustaining unhealthy eating and social behaviors in these patients, and should be adequately addressed during treatment, perhaps combining FBT and CBT approaches. Future studies with larger sample sizes and more detailed characterization of the study population are required to validate our conclusions. Moreover, study protocols specifically addressing the “language”, “perceptual-motor function”, and “complex attention” domains should also be designed, in order to complement the hereby proposed pathogenic model.

## Figures and Tables

**Table 1:** Main results from the selected studies.

Study	Participants	fMRI Paradigm	Main Findings	Main Conclusions
**EXECUTIVE FUNCTIONS**				
*Cognitive flexibility*				
Firk, 2015 [28]	AN *n* = 19 (females), subtype nd. Mean age 15.9 yrs (±1.5). Controls *n* = 20 (females). Mean age 15.9 yrs (±1.9).	Serial reaction time (SRT) Implicit learning	SRT performance was impaired in AN patients. AN patients also showed lower activity in the ventral anterior and ventral lateral thalamic nuclei compared with controls.	The impairment in cognitive flexibility in AN patients might contribute to the persistence of habitual behaviors (such as restricting the caloric intake) in these individuals.
Hildebrandt, 2018 [29]	AN *n* = 15 (females), subtype nd. Mean age nd. Controls *n* = 14 (females). Mean age nd.	Reversal learning	AN and controls did not differ on expectancy ratings (performance) on the task. During the association (learning) phase, AN had higher activity in the DLPFC, IFG, and IOG compared with controls. During the cue-reversal learning phase, AN showed greater activation in the VLPFC, DLPFC, IFG, and MOG compared with controls.	AN patients are as proficient as controls in reversing a stimulus-outcome learned association, however they require a greater engagement of top-down, inhibitory control regions. Thus, a higher cognitive control is required for AN patients compared with controls to achieve the same cognitive flexibility.
*Working memory*				
* Castro-Fornieles, 2010 [30]	AN *n* = 14 (12 females), subtype nd. Mean age 15.0 yrs (±1.7). Controls *n* = 14 (7 females). Mean age 15.4 yrs (±0.1).	1-back task	AN and controls did not differ in terms of performance (number of errors), however AN showed hyperactivity in the superior temporal gyrus during the task. They also had a trend for higher activity in several temporal and parietal areas, correlating positively with depressive symptomatology and negatively with BMI. Brain activity normalized weight restoration.	AN patients need more cognitive resources to achieve the same level of performance as controls. The cognitive load required is higher for individuals with higher depressive symptoms and lower BMI. Treatment and weight restoration can rescue cognitive abilities.
*Inhibitory control*				
Lock, 2011 [31]	AN *n* = 14 (females), restrictive subtype. Mean age 15.0 yrs (±1.7). Controls *n* = 13 (females). Mean age 15.9 yrs (±1.3).	Go-NoGo task	AN patients and controls did not differ on task accuracy. AN patients showed a positive correlation with percent correctly inhibited trials in the inferior parietal cortex, precuneus, and PCC.	In AN patients, successful response inhibition is associated with greater recruitment of brain regions underlying visual attention and visual working memory.
Wierenga, 2014 [32]	AN *n* = 11 (females), restrictive type. Mean age 16.0 yrs (±2.0). Controls *n* = 12 (females). Mean age 14.9 yrs (±1.8).	Stop signal task (SST)	AN patients had lower post-error slowing. AN patients had lower activity in the dorsal anterior ACC, MFG, and PCC during hard trials compared with controls. Patients also had lower activity in MFG and PCC during error (failed inhibit) processing trials, compared with controls.	AN patients have impaired representation of task difficulty, reflecting impaired cognitive flexibility. Nonetheless, they seem to require less resources for error monitoring.
**LEARNING AND MEMORY**				
*Reward processing*				
Bischoff-Grethe, 2013 [33]	AN *n* = 10 (females), restrictive subtype. Mean age 16.2 yrs (±1.8). Controls *n* = 12 (females). Mean age 15.4 yrs (±1.6)	Monetary guessing task	AN showed normal responses to reward in the anterior limbic system, but greater response to punishment compared with controls in the posterior caudate and in the cognitive cingulate cortex. Controls were more responsive to reward in the anterior putamen and motor cingulate cortex, compared with AN.	During action-outcome learning, AN patients have normal reward expectancies; however they are highly sensitive to punishment (negative feedback). This impairs their ability to appropriately proportion reward and punishment in order to learn from experience.
**SOCIAL COGNITION**				
*Emotional processing*				
Horndasch, 2018 [34]	AN *n* = 15 (females), subtype nd. Mean age 16.4 yrs (±1.4). Controls *n* = 18 (females). Mean age 15.9 yrs (±2.1).	Viewing and rating pictures of high-calorie food, low-calorie food, negative, neural, positive stimuli	No differences were found in the ratings of emotional stimuli. Controls showed greater activity compared with AN patients in the cerebellum, ACC, striatum, and inferior frontal gyrus for negative stimuli; in the cerebellum for neutral stimuli; in the cerebellum, striatum, precuneus, ACC, inferior frontal gyrus and hippocampus for positive stimuli. AN patients had higher activity than controls when viewing neutral and positive stimuli in the medial PFC.	AN patients showed lower processing of all emotional stimuli with some specific regions involved in positive picture processing, possibly reflecting impaired ability to experience pleasure by daily natural reinforcers.
*Theory of the Mind*				
* Schulte-Ruther, 2012 [35]	AN *n* = 19 (females), 13 restrictive subtype. Mean age 15.7 yrs (±1.5). Controls *n* = 21 (females). Mean age 15.8 yrs (±1.9).	Social attribution task (SAT)	AN patients and controls did not differ in the attribution of social relations. At baseline, AN patients had lower activity in the STG, MTG, and TP compared with controls when viewing social vs. non-social videos. After weight restoration, patients still had lower activity compared with controls in the MTG and TP. Lower baseline activity correlated with worse treatment outcome.	AN patients show impaired social functioning and social mentalization abilities, partially persisting after treatment and weight restoration. Poorer social cognition correlates with worse treatment outcome.
*Social evaluation*				
† Xu, 2017 [36]	AN *n* = 24 (females), 19 restrictive subtype. Mean age 16.4 yrs (±2.0). Controls *n* = 18 (females). Mean age 16.1 yrs (±2.3).	Social Identity-V2 task. Reading and responding (agree or disagree) to statements related to thinking about oneself, one’s friend, or what one’s friend thinks of them.	AN patients and controls did not differ on neural activity. Within patients, PCC activity was higher in response to friend-relative-to-self evaluations in recovered patients compared with those who remained ill. MPFC-dACC activity correlated with concerns about body shape, and MPFC-Cing activity correlated with anxiety levels.	Differences in social evaluations may contribute to both anxiety and body shape concerns in AN, and might have clinical predictive value.
**ED-RELATED STIMULI**				
*Body image perception*				
Fladung, 2013 [37]	AN *n* = 13 (females), 10 restrictive subtypes. Mean age 16.0 yrs (±1.1). Controls *n* = 14 (females). Mean age 16.6 yrs (±1.1).	Viewing and rating images of underweight, normal weight, and overweight female bodies.	AN patients rated underweight stimuli as more satisfying compared with controls. Controls had striatal higher activity compared to patients when processing normal-weight stimuli, while patients had higher striatal activity when processing underweight stimuli.	AN might engage the same circuitry involved in addiction disorders, and might thus be considered as a starvation dependence.
Seeger, 2002 [38]	AN *n* = 3 (females), subtype nd. Mean age 17.0 yrs (±0.5). Controls *n* = 3 (females). Mean age 17.5 yrs (±0.5).	Viewing: (1) distorted images of own body; (2) distorted imagesof another woman’s body;(3) scrambled images with mixed colors composed of own body images.	AN patients showed greater activity in the brainstem, right amygdala, and right gyrus fusiformis when viewing distorted own body image versus an average of non-target and neutral pictures.	AN patients show aversive responses when confronted with distorted images of own body shape.
Wagner, 2003 [39]	AN *n* = 13 (females), 10 restrictive subtype. Mean age 15.3 yrs (±1.4). Controls *n* = 10 (females). Mean age 15.1 yrs (±1.9).	Viewing: (1) distorted images of own body; (2) distorted images of another woman’s body; (3) scrambled images with mixed colors composed of own body images.	The PFC activity was higher for own body than for other women’s body or neutral pictures in controls, while it was higher in patients for both own body or other women’s body. AN patients had higher activity in the IPL when viewing own body compared with other women’s body or abstract shapes, while no differences were observed in controls.	AN patients might have an unspecific greater attention toward body stimuli, plus a specific visuo-spatial processing of own body shape.
*Food stimuli*				
Horndasch, 2018 [34]	AN *n* = 15 (females), subtype nd. Mean age 16.4 yrs (±1.4). Controls *n* = 18 (females). Mean age 15.9 yrs (±2.1).	Viewing and rating pictures of high-calorie food, low-calorie food, negative, neural, positive stimuli	AN patients gave lower ratings to high calorie foods compared with controls, while no differences were found in the ratings of low calorie foods. High-calorie foods elicited stronger IFG, medial prefrontal gyrus, and anterior insula activation in AN patients, but lower activity in the cerebellum compared with controls. For low-calorie stimuli, controls showed higher activity in the right cerebellum, and lower activity in the left cerebellar, medial PFC and parietal lobe compared with AN patients.	AN patients showed hyperactivity of the bottom-up and top-down areas in response to food.

* longitudinal study included acute and remitted patients; † study focused on weight-restored patients; ACC—anterior cingulate cortex; AN—anorexia nervosa; BMI—body mass index; Cing—cingulate cortex; dACC—dorsal anterior cingulate cortex; DLPFC—dorsolateral prefrontal cortex; IFG—inferior frontal gyrus; IOG—inferior occipital gyrus; IPL—inferior parietal lobule; MFG—middle occipital gyrus; MOG—middle occipital gyrus; MPFC—medial prefrontal cortex; MTG—middle temporal gyrus; nd—not defined; PCC—posterior cingulate cortex; PFC—prefrontal cortex; SRT—serial reaction time; SST—stop signal task; STG—superior temporal gyrus; TP—temporal pole; VLPFC—ventrolateral prefrontal cortex.

**Table 2:** Quality assessment of studies

Paper	Development, Demographic Data, and Illness State (0–13.5)	Effects of Exercise, Hydration Status, Binge Eating and Purging, and Malnutrition (0–16.25)	Stage of Treatment (0–6.0)	Hormonal Effects (0–9.25)	Comorbidity and Medication (0–10.0)	Technical and Statistical Considerations, and Study Design (0–15.25)	TOT (0–70.0)
Bischoff-Grethe, 2013	7.75	7	1.5	3	6	10.5	35.75
Castro-Fornieles, 2010	7.5	11.5	4	3	6.5	3	35.50
Firk, 2015	3	4	4	3	10	7.5	31.50
Fladung, 2013	6	4.5	0	3	0	4.5	18.00
Hildebrandt, 2018	0	0	0	3	5	4.5	12.50
Horndasch, 2018	6	7	2.5	3	6	4.5	29.00
Lock, 2011	9.25	6	1.5	6	3	7.5	33.25
Schulte-Ruther, 2012	3	6.5	4	3	6	9	31.50
Seeger, 2012	3	4	4	3	10	6	30.00
Wagner, 2003	3	5.5	4	3	8.5	6	30.00
Wierenga, 2014	6.25	10	4	3	3	10.5	36.75
Xu, 2017	6.25	3.5	4	6	3	7.5	30.25
**Mean score**	**5.1**	**5.8**	**2.8**	**3.5**	**5.6**	**6.75**	**29.50**

## Supplementary Materials

The following are available online at https://www.mdpi.com/2072-6643/11/8/1907/s1, Table S1: PRISMA Checklist.
