# Supplementary material for: Brain and Cognitive Development in Adolescents with Anorexia Nervosa: A Systematic Review of fMRI Studies
Source: Nutrients. 2019 Aug 15;11(8):1907. doi: 10.3390/nu11081907 (PMC6723243; doi:10.3390/nu11081907)
Supplement: Supplementary file 1 [file nutrients-11-01907-s001.pdf]

Table S1. PRISMA Checklist.

Section/Topic		Checklist Item	Reported on Page
**Title**			
Title	1	Identify the report as a systematic review, meta-analysis, or both.	1
**Abstract**			
Structured summary	2	Provide a structured summary including, as applicable: background; objectives; data sources; study eligibility criteria, participants, and interventions; study appraisal and synthesis methods; results; limitations; conclusions and implications of key findings; systematic review registration number.	1
**Introduction**			
Rationale	3	Describe the rationale for the review in the context of what is already known.	1–3
Objectives	4	Provide an explicit statement of questions being addressed with reference to participants, interventions, comparisons, outcomes, and study design (PICOS).	3
**Methods**			
Protocol and registration	5	Indicate if a review protocol exists, if and where it can be accessed (e.g., Web address), and, if available, provide registration information including registration number.	n/a
Eligibility criteria	6	Specify study characteristics (e.g., PICOS, length of follow-up) and report characteristics (e.g., years considered, language, publication status) used as criteria for eligibility, giving rationale.	3
Information sources	7	Describe all information sources (e.g., databases with dates of coverage, contact with study authors to identify additional studies) in the search and date last searched.	3
Search	8	Present full electronic search strategy for at least one database, including any limits used, such that it could be repeated.	3–4
Study selection	9	State the process for selecting studies (i.e., screening, eligibility, included in systematic review, and, if applicable, included in the meta-analysis).	3–4
Data collection process	10	Describe method of data extraction from reports (e.g., piloted forms, independently, in duplicate) and any processes for obtaining and confirming data from investigators.	3–4
Data items	11	List and define all variables for which data were sought (e.g., PICOS, funding sources) and any assumptions and simplifications made.	3–5
Risk of bias in individual studies	12	Describe methods used for assessing risk of bias of individual studies (including specification of whether this was done at the study or outcome level), and how this information is to be used in any data synthesis.	4–5
Summary measures	13	State the principal summary measures (e.g., risk ratio, difference in means).	3–4
Synthesis of results	14	Describe the methods of handling data and combining results of studies, if done, including measures of consistency (e.g., I^2) for each meta-analysis.	n/a
Risk of bias across studies	15	Specify any assessment of risk of bias that may affect the cumulative evidence (e.g., publication bias, selective reporting within studies).	4–5
Additional analyses	16	Describe methods of additional analyses (e.g., sensitivity or subgroup analyses, meta-regression), if done, indicating which were pre-specified.	n/a
**Results**			
Study selection	17	Give numbers of studies screened, assessed for eligibility, and included in the review, with reasons for exclusions at each stage, ideally with a flow diagram.	4–5 (Figure 2; Table 1)

Study characteristics	18	For each study, present characteristics for which data were extracted (e.g., study size, PICOS, follow-up period) and provide the citations.	14–17 (Table 1)
Risk of bias within studies	19	Present data on risk of bias of each study and, if available, any outcome level assessment (see item 12).	17 (Table 2)
Results of individual studies	20	For all outcomes considered (benefits or harms), present, for each study: (a) simple summary data for each intervention group (b) effect estimates and confidence intervals, ideally with a forest plot.	(a) 14–17 (Table 1); (b) n/a
Synthesis of results	21	Present results of each meta-analysis done, including confidence intervals and measures of consistency.	n/a
Risk of bias across studies	22	Present results of any assessment of risk of bias across studies (see Item 15).	17 (Table 2)
Additional analysis	23	Give results of additional analyses, if done (e.g., sensitivity or subgroup analyses, meta-regression [see Item 16]).	n/a
**Discussion**			
Summary of evidence	24	Summarize the main findings including the strength of evidence for each main outcome; consider their relevance to key groups (e.g., healthcare providers, users, and policy makers).	24–25 (Figures 3 and 4)
Limitations	25	Discuss limitations at study and outcome level (e.g., risk of bias), and at review-level (e.g., incomplete retrieval of identified research, reporting bias).	25–26
Conclusions	26	Provide a general interpretation of the results in the context of other evidence, and implications for future research.	26
**Funding**			
Funding	27	Describe sources of funding for the systematic review and other support (e.g., supply of data); role of funders for the systematic review.	26

From Moher D, Liberati A, Tetzlaff J, Altman DG, The PRISMA Group (2009). Preferred Reporting Items for Systematic Reviews and Meta-Analyses: The PRISMA Statement. PLoS Med 6(7): e1000097. doi:10.1371/journal.pmed1000097.

Quality Assessment

DEVELOPMENT, DEMOGRAPHIC DATA, AND ILLNESS STATE

1 (*3). Study groups should be age matched, and age may need to be included as a covariate in between-group analyses. (1 = age matched; 0 = not age-matched. |+ 0.25 if age was included as covariate in the analysis)

2 (*1). Race and ethnicity should be considered, either by matching groups or factoring these characteristics into analyses. (1 = matched for ethnicity or ethnicity considered in the analysis; 0 = not matched for ethnicity, and ethnicity not considered in the analysis)

3 (*3). Duration of illness (DI) should be reported and its effects on study results tested. (1 = DI reported; 0 = DI not reported |+ 0.25 if DI was tested against study results)

4 (*3). Handedness should be assessed, and either only right-handed individuals should be included or handedness should be accounted for in the data analysis. (1 = only right-handed individuals included or handedness accounted for in the analysis; 0 = mixed sample and handedness not considered in the analysis, or handedness not reported)

5 (*3). The effects of height-adjusted weight (body mass index - BMI) on brain measures should be tested. In youth, an age adjusted BMI percentile or BMI standard deviation scores (SDS) should be

reported. (1 = BMI/BMI-SDS per age reported and tested; 0.5 = BMI, not age-adjusted, reported and tested; 0 = BMI/BMI-SDS not tested)

6. Every effort should be made to describe level of recovery (duration, BMI history, eating disorder psychopathology) in a study sample and its relationship with brain findings. (n/a)

EFFECTS OF EXERCISE, HYDRATION STATUS, BINGE EATING AND PURGING, AND MALNUTRITION

7 (*3). The methods and procedures should indicate under what nutritional conditions a study was conducted, whether study participants controlled their own food intake or whether they were in a structured program where they had to follow a dietician's directed meal plan adjusted for nutritional needs. (1 = standardized eating schedule or inpatients treatment; 0.5 = eating schedule reported but not standardized or outpatients treatment; 0 = information not reported)

8 (*3). Binge-eating and purging frequency should be reported and tested against study outcome measures. (1 = homogeneous sample (all restrictive/all purging), or subtypes reported and tested; 0.5 = subtypes reported but not tested; 0 = subtypes not reported)

9 (*2). The methods should indicate whether patients have lost or gained weight or BMI in the weeks prior to brain imaging. (1 = recent weight history reported; 0 = recent weight history not reported)

10 (*3). Food intake prior to brain imaging should be standardized within studies (e.g., an 8 h fast or a standardized meal) and reported. (1 = standardized food intake prior to imaging procedure; 0.5 = food intake not standardized but tested; 0 = food intake not standardized or not reported)

11 (*1). Exercise should be quantified and the effects tested on brain imaging data. This is especially important for studies that recruit individuals who are not in a treatment program. The amount of exercise over the past 24 h prior to the study should be reported. Habitual exercise may also be of interest as it affects brain structure and function and should be reported and tested for effects on brain function or structure if available. (1 = exercise reported and tested, or inpatients treatment; 0 = exercise not reported)

12 (*3). Whether an individual has a history of an additional eating disorder other than the type described in a particular study should also be recorded and its effects on brain measures tested. (1 = history reported; 0 = history of other EDs not recorded |+ 0.25 if past EDs effect was tested)

13 (*1). We should specifically test to what extent regional brain structure measures, such as gray or white matter volume, may contribute to regional functional imaging findings. (1= brain volumes accounted for; 0 = brain volumes not accounted for)

STAGE OF TREATMENT

14 (*3). Publications should describe whether study participants were in a specific treatment setting or not. (1= treatment described or patients not in treatment; 0.5 = patients were in treatment, but treatment was not described; 0 = information not reported)

15 (*1). Studies should indicate whether study participants that were in treatment were all in the same setting or treated with the same treatment modality, or not. (1 = all treated in the same setting/with same modality; 0.5 = treated with different modalities; 0 = information not reported)

16 (*2). The length of time in treatment and BMI change between starting treatment and scanning should be reported. (1 = length of treatment and BMI changes reported; 0 = information not reported)

HORMONAL EFFECTS

17 (*3). Given the effects of sex hormones on the brain, sex must be considered and, when possible, males and females studied separately. (1 = only one sex included or adjusted for sex, or analyses

performed separately; 0 = mixed sample, and analyses not adjusted for sex | + 0.25 if age was included as covariate in the analysis)

18 (*3). Menstrual status (e.g., pre- vs. post-menarcheal, presence, and duration of amenorrhea) should be documented and used in the analyses. (1 = menstrual status documented and used in the analyses; 0.5 = menstrual status documented but not used in the analyses; 0 = information not reported)

19 (*2). In menstruating females, imaging should ideally be done during the same menstrual cycle phase for all study participants (e.g., the early follicular phase when estradiol and progesterone levels are low). If conducting brain imaging during the same cycle phase is too impractical and difficult to undertake, the cycle phase (or sex hormone levels) should be recorded and taken into consideration when analyzing the brain imaging data. (1 = scans acquired in same cycle phase or cycle phase accounted for in the analysis; 0 = information not collected)

20 (*1). If possible, females should be studied at least eight weeks off of oral contraceptive pills (OCPs). Alternatively, if taking OCPs, females can be when compared with a control population also taking OCPs. If these approaches are not possible, then OCP use can be used as a covariate in analyses. (1 = eight weeks off of oral contraceptives or matched samples; 0.5 = analysis controlled for OCPs use; 0 = information not reported)

21. If hormone levels are measured, similar to the recommendation to standardize nutritional status, the conditions under which hormones are assessed (e.g., early morning and fasting) should be standardized and reported. (n/a)

22. Other factors that influence the hormonal milieu, such as exercise, sleep patterns, the level of stress, drugs, or supplements, and medical and psychiatric comorbidities, should be considered. (n/a)

COMORBIDITY AND MEDICATION

23 (*3). Study participants should be assessed using diagnostic instruments that go beyond the eating disorder diagnosis and those conditions should be reported. (1 = comorbidities assessed and instruments reported; 0.5 = comorbidities assessed but instruments not reported; 0 = comorbidities not assessed)

24 (*2). If a study sample includes individuals with comorbid conditions, the effects of these conditions on the brain imaging results should be assessed. (1 = no comorbidities, or comorbidities tested; 0 = presence of comorbidities, but not tested)

25 (*3). Any substances and alcohol for the past 24 h prior to the study should be recorded and reported and the effects on brain data assessed. Habitual use of substances over the past 3 months prior to the study are also of interest and should be reported if available. (1 = no substance use 24 h prior to scanning or inpatients treatment or substance use reported; 0.5 = substance use controlled for in the analysis; 0 = substance use not assessed)

26 (*2). Ideally, medication use should be stable for four to five half-lives before brain imaging to accomplish at least steady state conditions. (1 = no use of medications, or stable levels; 0 = use of medications of unspecified duration)

TECHNICAL AND STATISTICAL CONSIDERATIONS, AND STUDY DESIGN

27 (*3). Sample size and statistical analysis methods should be well justified. Some have suggested minimum numbers of study participants per group to capture the most prominent findings (Thirion et al., 2007). However, there is no standard approach and methods should describe what measures were taken to avoid Type I or Type II errors, including correction for multiple comparisons (Carter, 2016). (1 = multiple testing correction or cluster-extent thresholding applied, sample size > 20 individuals; 0.5= multiple testing correction or cluster-extent thresholding applied, sample size < 20 individuals or multiple testing correction or cluster-extent thresholding not applied, sample size > 20

individuals; 0 = no multiple testing correction or cluster-extent thresholding applied, sample size < 20 individuals)

28 (*3). Task stimuli in fMRI should be well matched with their respective control condition. (1= yes; 0 = no)

29 (*3). Studies should ensure that outliers, for instance due to extremely altered brain structure, do not drive group results. (1 = outliers checked for; 0 = outliers not assessed)

30 (*3). Whether data are normally distributed should be assessed and the appropriate test selected for statistical analysis. (1 =data checked for normality of the distribution; 0 = data not checked for normality of the distribution)

31 (*3). Describe in detail how motion correction was conducted. (1 = motion correction performed, excessive movement excluded and/or motion parameters included as 1st level covariates; 0 = realignment performed, but excessive movement not excluded and motion parameters not included as 1st level covariates)

References

Carter, C.S. As the field matures We begin. *Biol. Psychiatry Cogn. Neurosci. Neuroimaging* **2016**, *1*, 3–4.

Thirion, B.; Pinel, P.; Meriaux, S.; Roche, A.; Dehaene, S.; Poline, J.B. Analysis of a large fMRI cohort: Statistical and methodological issues for group analyses. *NeuroImage* **2007**, *35*, 105–120.
